# Resistance exercise and breast cancer–related lymphedema—a systematic review update and meta-analysis

**DOI:** 10.1007/s00520-020-05521-x

**Published:** 2020-05-15

**Authors:** Timothy Hasenoehrl, Stefano Palma, Dariga Ramazanova, Heinz Kölbl, Thomas E. Dorner, Mohammad Keilani, Richard Crevenna

**Affiliations:** 1 Department of Physical Medicine, Rehabilitation and Occupational Medicine, Medical University of Vienna, Waehringer Guertel 18-20, 1090 Vienna, Austria; 2 Section of Medical Statistics, CeMSIIS, Medical University of Vienna, Vienna, Austria; 3 Division for General Gynaecology and Gynaecological Oncology, Department of Gynaecology and Obstetrics, Medical University of Vienna, Vienna, Austria; 4 Department of Social and Preventive Medicine, Centre for Public Health, Medical University of Vienna, Vienna, Austria; 5 Versicherungsanstalt öffentlich Bediensteter, Eisenbahnen und Bergbau, Vienna, Austria

**Keywords:** Breast neoplasm, Breast cancer survivors, Resistance training, Secondary lymphedema, Strength, Medical training therapy, Lymphedema assessment

## Abstract

**Background:**

The purpose of this systematic review update and meta-analysis was to analyze resistance exercise (RE) intervention trials in breast cancer survivors (BCS) regarding their effect on breast cancer-related lymphedema (BCRL) status and upper and lower extremity strength.

**Methods:**

Systematic literature search was conducted utilizing PubMed, MEDLINE, and Embase databases. Any exercise intervention studies—both randomized controlled and uncontrolled—which assessed the effects of RE on BCRL in BCS in at least one intervention group published between 1966 and 31st January 2020 were included. Included articles were analyzed regarding their level of evidence and their methodological quality using respective tools for randomized and nonrandomized trials of the Cochrane collaboration. Meta-analysis for bioimpedance spectroscopy (BIS) values as well as upper and lower extremity strength was conducted.

**Results:**

Altogether, 29 studies were included in the systematic review. Results of six studies with altogether twelve RE intervention groups could be pooled for meta-analysis of the BCRL. A significant reduction of BCRL after RE was seen in BIS values (95% CI − 1.10 [− 2.19, − 0.01] L-Dex score). Furthermore, strength results of six studies could be pooled and meta-analysis showed significant improvements of muscular strength in the upper and lower extremities (95% CI 8.96 [3.42, 14.51] kg and 95% CI 23.42 [11.95, 34.88] kg, respectively).

**Conclusion:**

RE does not have a systematic negative effect on BCRL and, on the contrary, potentially decreases it.

**Electronic supplementary material:**

The online version of this article (10.1007/s00520-020-05521-x) contains supplementary material, which is available to authorized users.

## Introduction

Breast cancer is the most common cancer in women with incidence rates of over a quarter million new cases in the USA [1–3] and just short of half a million in Europe [4] representing about one third of all new cancer diagnoses in women [3]. Of those patients, about 20% develop breast cancer–related lymphedema (BCRL) over the course of their treatment [5]. Lymphedema is an excess accumulation of a protein-rich fluid which would otherwise drain through the lymphatic system and leads to a regional swelling—in the particular case of BCRL—the swelling of the arm of the affected side [6]. It is associated with symptoms like pain, heaviness, tightness, decreased range of motion, adversely affected gross and fine motor skills, impaired daily function, and decreased quality of life [7, 8]. BCRL is currently considered being an incurable and chronic disease and its treatment aims on the management of the lymphedema status and the preservation of the function of the affected arm [9]. Up-to-date standard of care is the complete decongestive therapy consisting of manual drainage, bandaging, compression, skin care, and exercise [9].

Exercise as a part of the current treatment standard of care is a more recent development. Up to the early 2000s, breast cancer survivors (BCS) were advised to refrain from “vigorous, repetitive, or excessive upper body exercise” because of the fear that these physical activities could lead to the development of a new or an increase of an existing lymphedema [10]. Only some decades ago, Harris and Niesen-Vertommen [10] started to challenge the myth that intensive physical loading of the affected arm side of BCS would lead to either the development of a fresh or the increase of an already existing BCRL. This field of research they initiated then has led to publication of over twenty resistance exercise (RE) intervention studies and a number of systematic reviews [11–13], and it is safe to say that the initial fears that physical loading could harm the BCS via negatively affecting the BCRL were unjustified. Moreover, there is agreement that BCS will benefit from RE through maintaining and regaining physical function of their affected arm as well as a healthy body composition and therefore reducing metabolic risk [11, 12]. However, removing the fear from and changing an old paradigm in the heads of all health care professionals—starting with all involved physicians over the nursing staff to the physical and occupational therapists—so basically everyone a breast cancer patient will be involved with during her treatment, is a tough challenge. One reason for this might be that until this day any summaries of the existing trials were limited by the fact that yet no gold standard measurement method for the assessment of BCRL has been established. Therefore, the only existing meta-analysis which tried to pool the results of the then existing literature regarding the lymphedema status after a RE intervention was forced to pool the results of different lymphedema assessment techniques [14]. Till today, the lack of a gold standard measurement method for the assessment of BCRL has prevented the conduction of a thorough meta-analysis, as the results of at least five exercise intervention studies assessing BCRL with the same assessment method are necessary to assure reliability when a small number of heterogeneous studies are used [15].

Therefore, the aims of the current study are to, on the one hand, give an update over the current literature regarding RE in BCS and, on the other hand, to perform a meta-analysis of the lymphedema status if the systematic literature review might show sufficient homogeneous BCRL assessment results present.

## Methods

A systematic literature review was conducted using the scientific databases PubMed, Embase, and MEDLINE between 1966 and 31st January 2020. The search strategy included the search terms “lymphedema,” “lymphoedema” AND “breast cancer” AND “resistance exercise,” “resistance training,” “strength exercise,” “weight training,” “weight lifting,” and their possible variations. Any exercise intervention studies—both randomized controlled and uncontrolled—which assessed the effects of RE on BCRL in BCS in at least one intervention group published between 1966 and 31st January 2020 and followed or exceeded the RE intensity recommendations of the American College of Sports Medicine (ACSM) for BCS [16, 17] were considered. Moreover, assessment of the BCRL with any assessment technique and English language were deemed mandatory for being considered for inclusion. During the systematic literature research, 747 articles were found and checked for title and abstract. Of those, 46 were chosen for full-text analysis, while 701 were excluded right after analysis of the title and abstract. After full-text analysis, 29 articles fulfilled the inclusion criteria and were therefore included in the systematic review [18–46]. An overview over the selection process is presented in Supplementary Fig. 1. The process of systematic literature review as well as selection of suitable articles was conducted independently by two experienced researchers following the PRISMA reporting guidelines for systematic reviews and meta-analyses [47].

As the current article is a review update of former systematic reviews [11, 12] and exactly the same search strategy and inclusion as well as exclusion criteria were applied, the main focus of the systematic review was on the articles published since 30th September 2017, the end of the last literature search [11]. Six new articles were found [41–46] which were introduced to risk of bias analysis. Five of those articles were randomized studies [41–44, 46] and were therefore assessed with the current risk of bias tool for randomized trials of the Cochrane collaboration, the RoB 2 [48]. The sixth one, Luz et al. [45], however, was a nonrandomized trial. Therefore, the risk of bias analysis for this study was undertaken with the Risk Of Bias In Non-randomized Studies of Interventions (ROBINS-I) tool of the Cochrane collaboration [49].

All of the included studies were then checked for their LE outcome assessment and if the outcome assessment and the presentation of the results were homogeneous enough for being suitable for a meta-analysis [15]. All outcome variables measured with the same assessment method in five or more of the original studies were subject to meta-analyses. Outcome variables reported in less than five studies were not considered for meta-analysis as the results are considered unreliable when a small number of heterogeneous studies are used. Moreover, studies which performed their LE assessment with circumference measurements were disregarded due to high variability in measurement technique.

### Statistical analyses

The primary endpoint of this meta-analysis is the average difference of measurements before and after training. Some authors did report the average differences as well as their standard deviations. For the studies which lacked this information, the mean differences were calculated with simple subtraction (“mean.follow-up” − “mean.baseline”). The standard deviations were calculated via confidence intervals (i.e., a two-sided, confidence interval for a paired sample mean difference from a normal distribution with unknown variance). Other missing standard deviations were estimated using the average correlation of other studies. The statistical analyses were calculated using meta-analyses with a random intercept for each study. The models were fitted via restricted maximum-likelihood (“REML”) estimation; test statistics and confidence intervals for the fixed effects were computed based on *t*-distribution. All statistics were conducted using package metafor, R (version 3.6).

## Results

### Level of evidence and risk of bias analysis

Levels of evidence as well as the type of study design are depicted in Table 1. Of the newly included studies [41–46], all but Luz et al. [45] were classified 1b and can therefore be ranked high in the hierarchy of evidence.

**Table 1:** Level of evidence and study design of the included studies published since 30th September 2017 and details of previous studies published in Hasenoehrl et al. [11] and Keilani et al. [12]

Study	Level of evidence	Study design
Ammitzbøll et al. [41]	1b	Randomized controlled trial
Bloomquist et al. [42]	1b	Randomized, crossover, equivalence trial
Bloomquist et al. [43]	1b	Randomized controlled trial
Luz et al. [45]	2b	Controlled clinical trial
Omar et al. [44]	1b	Single-blinded randomized controlled trial
Schmitz et al. [46]	1b	Randomized controlled clinical trial

*Comp* compression, *CPT* complex physical therapy, *Cont* control group, *Exerc* exercise, *HL* high load, *LL* low load, *ML* moderate load, *RE* resistance exercise, *ST* strength training

### Risk of bias analysis

The risk of bias analysis showed good methodological quality in the randomized trials [41–44, 46] with overall low risk of bias in three articles [41, 42, 46] and moderate risk of bias in two articles [43, 44] (Supplementary Fig. 2). The only nonrandomized trial showed serious overall risk of bias [45] (Supplementary Table 1).

### Patients and exercise details

Patients and exercise details can be found in aggregated form in Table 2. To complete Table 2, more detailed information regarding the exercise intervention was derived from two earlier articles [50, 51].

**Table 2:** Patient details, lymphedema status, and exercise details of the independent studies published since September 2017 and details of previous studies published in Hasenoehrl et al. [11] and Keilani et al. [12]

Study	Year	Sample	Patient details/LE status	Exercise duration, frequency, intensity	Exercise details	Compression during RE
Ammitzbøll et al. [41]	2019	32 PRE: *n* = 16 Cont: *n* = 16	Patients undergoing BCa surgery with axillary lymph node dissection	*Duration*: 50 weeks: 20 weeks supervised + 30 weeks self-administered *Frequency*: 3 times/week *RE intensity*: Progressing from 25 RM to 10–12 RM	*Sets*: 2–3 *Rep/set*: Weeks 1–4: 15–20 repetitions at the 25 RM, 2–3 sets Weeks 5–8: 15–17 repetitions at the 20 RM, 3 sets Weeks 9–12: 10–12 repetitions at the 15 RM, 3 sets Weeks 13–50: 10–12 repetitions at the 10–12 RM *Muscle groups*: Major muscle groups for upper limb, lower limb, and core *Exercises used*: Minimum of six exercises per session: three for the upper body, one for the lower body, and two for the core	Yes, if deemed necessary
Bloomquist et al. [42]	2018	18 in cross-over design RE-HL: *n* = 18 RE-LL: *n* = 17	Women receiving standard adjuvant chemotherapy for stage I–III BCa	*Duration*: 2 single sessions over 2 weeks *Frequency*: 1 RE session – 7 days washout – 1 RE session *RE intensity*: RE-HL: 85–90% 1RM RE-LL: 60–65% 1RM	*Rep/set:* RE-LL: 2 sets of 15–20 repetitions RE-HL: 3 sets of 5–8 repetitions *Exercises/muscle groups:* Chest press, latissimus pulldown and triceps extension with exercise machines, biceps curls with free weights	No
Bloomquist et al. [43] Additional information derived from Bloomquist et al. [43]	2019	153 High: *n* = 75 Low: *n* = 78	Physically inactive women receiving adjuvant chemotherapy for BCa	*Duration*: 12 weeks *Frequency*: 3 times/week *RE intensity*: High: 85–90% 1RM Low: no RE	*Rep/set*: High: Weeks 1–6: RE + AE + relaxation + massage Week 1: 8–12 repetitions at 70% 1RM, 2–3 sets Week 2: 8–12 repetitions at 80% 1RM, 2–3 sets Weeks 3–12: 5–8 repetitions at 80–90% 1RM, 2–3 sets Weeks 7–12: RE + AE + ballgames + dancing *Exercises/muscle group*: Major muscle groups of the body: leg press, chest press, latissimus pull down, abdominal crunch, lower back and knee extension Low: walking + health consultation	No information
Luz et al. [45]	2018	42 CPT: *n* = 22 CPT + ST: *n* = 20	BCS diagnosed with LE resulting from unilateral surgery for BCa treatment	*Duration*: 8 weeks *Frequency*: 2 times/week *RE intensity*: CPT: no RE CPT + ST: 40% 1RM	*Rep/set*: CPT: therapeutic exercises CPT+ST: Week 1: 10 repetitions, 2 sets Week 2: 10 repetitions, 3 sets Weeks 3–8: 15 repetitions, 3 sets *Exercises/muscle groups*: Shoulder abduction, elbow extension, external and internal rotation with resistance band, protraction/retraction of the shoulder blades with a stick, shoulder flexion and abduction, elbow flexion, fist flexion and extension with a sling, ball pressing and moving	Yes, part of the CPT
Omar et al. [44]	2019	70 RE + comp: *n* = 35 RE: *n* = 35	Women with unilateral BCRL and ≥ 5% of interlimb differences of volume or circumference	*Duration*: 8 weeks *Frequency*: 3 times/week *RE intensity*: 50–60% est1RM	*Rep/set*: 10–12 repetitions, 2–3 sets *Exercises/muscle groups*: Dumbbell fly, triceps extension, biceps curl up, one-arm bent over row, dumbbell sides rise, lifting the arm forward, and wrist curls with dumbbells	Yes, depending on personal preference
Schmitz et al. [46] Additional information derived from Winkels et al. [51]	2019	351 Cont: *n* = 90 Exerc: *n* = 87 Weight loss: *n* = 87 Exerc + weight loss: *n* = 87	Overweight BCS with BCRL	*Duration*: 52 weeks *Frequency*: 2 times/week *RE intensity*: no information	*Rep/set*: Exerc and exerc + weight loss Weeks 1–6: 1 supervised session (exercise instruction) + 1 unsupervised Weeks 1–4: 10 repetitions, 2 sets Weeks 7–52: 2 home-based exercise sessions, 1 weekly support telephone call + 1 monthly in-person class Weeks 5–52: 10 repetitions, 3 sets *Exercises/muscle groups*: Chest-presses, squats on a chair, one-arm rowing exercise, side-raises, step-ups, kickbacks, split-leg lunges, side lunges, and bicep curls with adjustable dumbbells + core training exercises of abdominal and lower back muscles (1 stabilization, 1 flexion, and 1 extension core exercise)	Yes

*AE* aerobic exercise, *BCa* breast cancer, *BCRL* breast cancer related lymphedema, *BCS* breast cancer survivor, *Comp* compression, *Cont* control group, *CPT* complex physical therapy, *est1RM* estimated 1-repetition-maximum, *Exerc* exercise, LE lymphedema, *PRE* progressive resistance exercise, *Rep/set* repetitions per set, *RE* resistance exercise, *RE-LL* low load resistance exercise, *RE-HL* high load resistance exercise, *ST* strength training, *RM* repetition-maximum

### Lymphedema assessment

Details of the LE assessment can be found in aggregated form in Table 3. To complete Table 3, more details regarding the calculation of limb volume from circumference measurements were derived from Taylor et al. [52].

**Table 3:** Lymphedema assessment, measurement details, and outcomes of the 6 newly included articles [41–46] published since September 2017 and details of previous studies published in Hasenoehrl et al. [11]

Author	Lymphedema assessment	Measurement details	Results
Ammitzbøll et al. [41]	Water displacement DXA	No measurement details Lymphedema was defined as a > 3% increase in ILVD. Measured outcome: ILMD Separate arm scans analyzed with Small Animal Program software (version 8.1027). In the subgroup of one study center (*n* = 77)	No significant mean change in ILVD No significant mean change in ILMD
Bloomquist et al. [42]	BIS DXA	Impedance of the extracellular fluid in the affected and nonaffected arms was assessed and compared (L-Dex score). Tissue composition and arm volume using a three-compartment model that is sensitive to changes in upper extremity tissue composition Using previously derived densities for fat (0.9 g mL^−1^), lean mass (1.1 g mL^−1^), and bone mineral content (1.85 g mL^−1^), DXA measurements were converted into estimated arm volumes.	Predetermined equivalence margin of ± 3.0 units: Equivalence between intensities was observed immediately after and 24 h after RE sessions. At 72 h post-RE session, equivalence could not be declared (lower CI exceeded − 3.0) favoring heavy load RE. Equivalence between intensities was observed at all time points for interlimb volume percent differences.
Bloomquist et al. [43]	DXA BIS	Equal to Bloomquist et al. [42] Equal to Bloomquist et al. [42] From participant 71 forward (*n* = 81)	Predetermined equivalence margin of ± 3.0 units: Nonequivalence was observed at all time points for interarm volume % differences favoring the HI-RE group. Equivalence between groups at 12 and 39 weeks. Equivalence to the predetermined equivalence margin at 12 weeks (per-protocol analysis) Nonequivalence to the predetermined equivalence margin at 39 weeks (upper CI exceeded 3.0) favoring the HI-RE group
Luz et al. [45]	Arm circumference	Measurement sites: • 14 and 7 cm above the olecranon • Circumference of the olecranon • 7, 14, and 21 cm below the olecranon • Circumference of the dorsum and palm, at the line of the metacarpals at the base of the fingers Further details: • Limb volume was calculated with the formula: *V* = *h*(*C*_1_^2^ + *C*_1_*C*_2_ + *C*_2_^2^)/(12π) [52] • *V* is the volume of the limb segment, *C* and *c* are the circumferences at each end, and *h* is the distance between the circumferences (*C*).	Between group change in arm volume not significant Within-group change showed decreased values in both RE groups (no level of significance reported).
Omar et al. [44]	Arm circumference	Measurement sites: • Circumference was taken at the levels of metacarpal and wrist, and at 4-cm intervals up the arm until the base of the axilla for both affected and unaffected limbs Further details: • Limb volume was calculated with the formula: *V* = *h*(*C*_1_^2^ + *C*_1_*C*_2_ + *C*_2_^2^)/(12π) [52]	At the end of treatment (week 8), the ELV and %ELV decreased significantly in both groups. These reductions were sustained to follow-up (week 12). No significant changes in the relative volume (% reduction ELV) were observed between groups at the end of treatment (week 8) or at follow-up (week 12).
Schmitz et al. [46]	Arm volume (perometry)	Outcome measure: percentage of interlimb volume differences	No between-group differences were noted at baseline or in 12-month changes in percentage or absolute interlimb differences. Individual limb decreases across 12 months were larger for both affected and unaffected limbs in the weight loss and combined intervention groups compared with the control group.

*BIS* bioimpedance spectroscopy, *CI* confidence interval, *DXA* dual X-ray absorptiometry, *ELV* excess limb volume, *HI* high intensity, *ILMD* interlimb mass difference, *ILVD* interlimb volume difference, *RE* resistance exercise

### Meta-analyses of lymphedema (BIS)

**Fig. 1:**
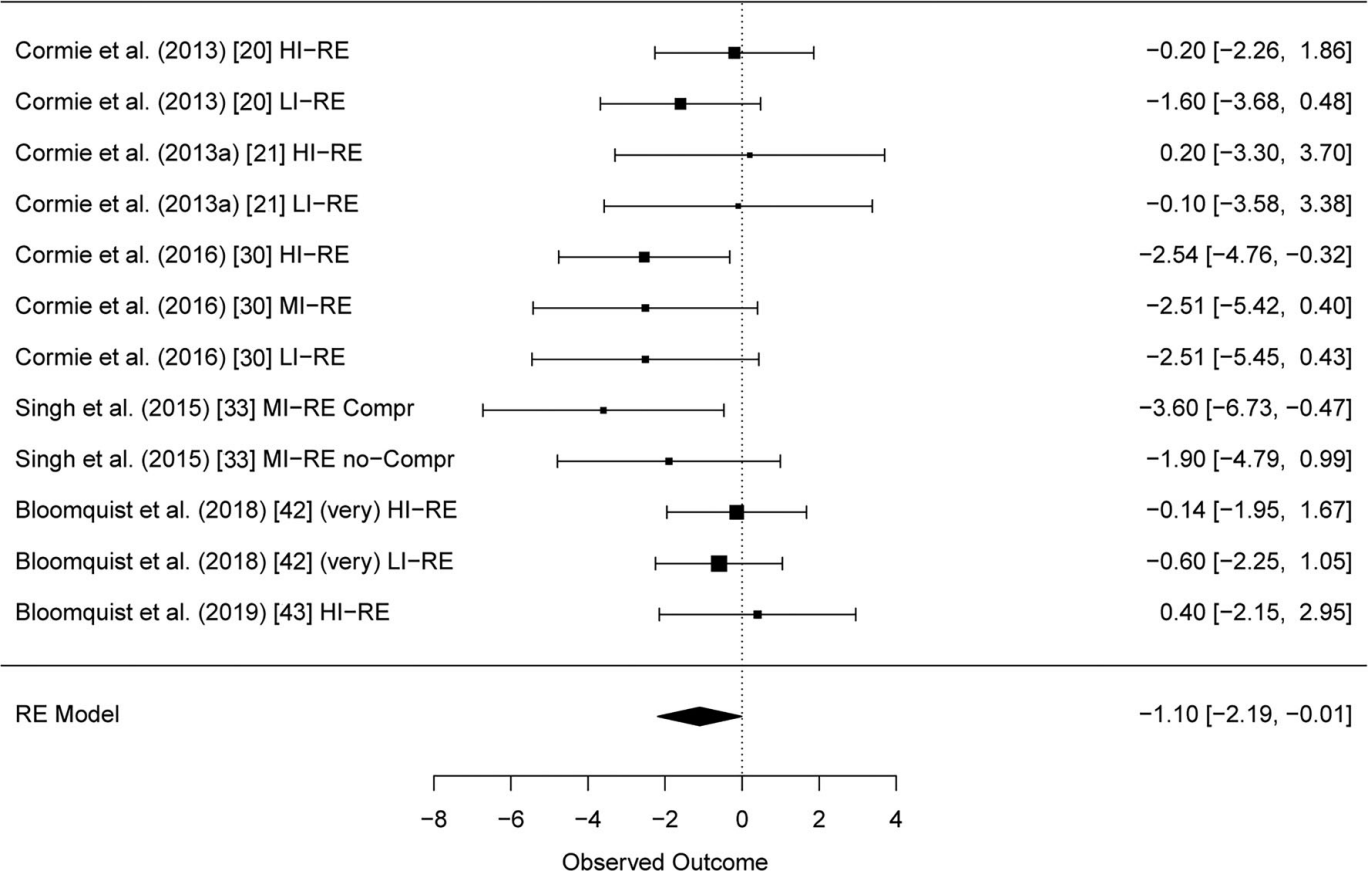
Forest plot bioimpedance spectroscopy (BIS)

After thorough analysis of the reported LE data in all included studies, the results of six RE intervention studies [20, 21, 30, 33, 42, 43] could be pooled for a meta-analysis. All of those studies assessed BCRL with BIS and reported L-Dex values. Test for heterogeneity was not significant, and therefore, homogeneity between the studies can be assumed (*Q*(*df* = 11) = 10.7104, *p* = 0.4678). The mean differences as well as their standard errors are presented in Supplementary Table 2. As indicated in Fig. 1, RE was associated with a significant decrease in L-Dex values (95% CI − 1.10 [− 2.19, − 0.01]). The funnel plot for BIS showed no sign of publication bias (Supplementary Fig. 3).

### Meta-analyses of upper extremity strength

**Fig. 2:**
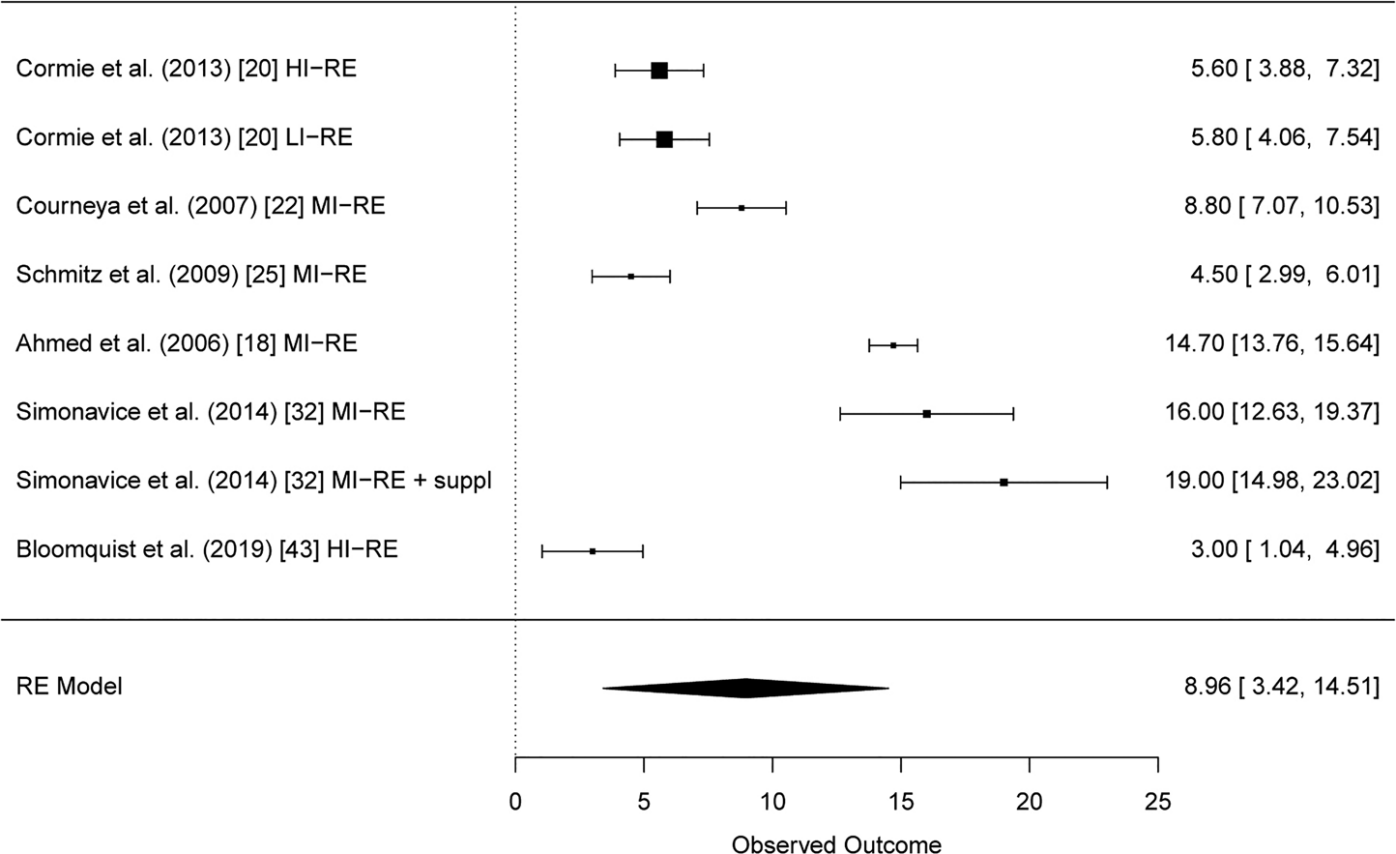
Forest plot upper extremity strength (chest press)

After thorough analysis of the reported strength data in all included studies, the results for upper extremity strength (chest press) could be pooled from six studies [18, 20, 22, 25, 32, 43]. Test for heterogeneity of upper extremity strength was significant which implies heterogeneity between the studies (*Q*(*df* = 7) = 275.37, *p* < 0.0001). The mean differences as well as their standard errors are presented in Supplementary Table 3. The meta-analysis model for upper extremity strength showed significant higher strength values after RE (95% CI 8.96 [3.42, 14.51]) (Fig. 2). The funnel plot for upper extremity strength showed no sign of publication bias as the observed outcome is evenly distributed around the average (Supplementary Fig. 4).

### Meta-analyses of lower extremity strength

**Fig. 3:**
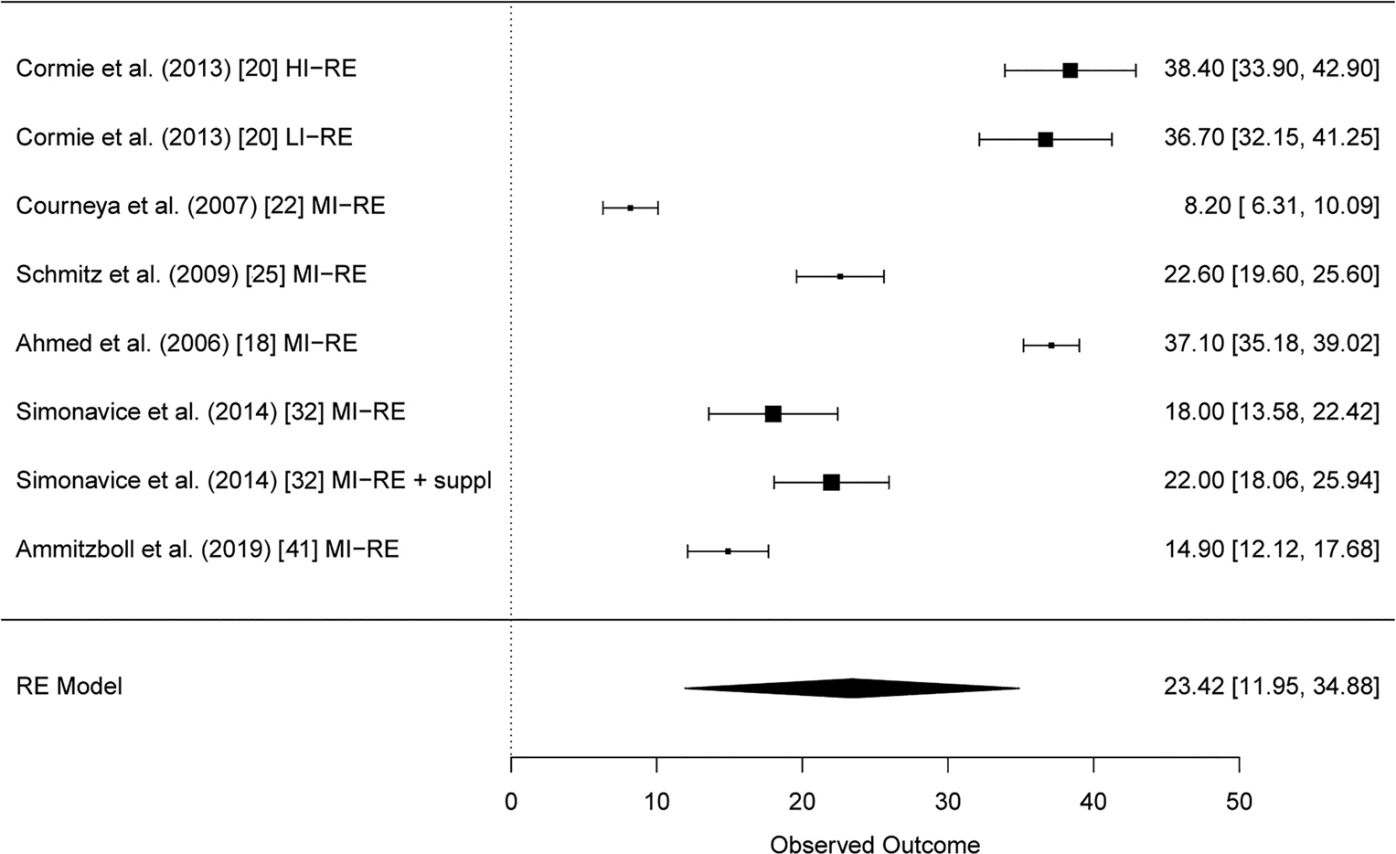
Forest plot lower extremity strength (leg press and extension)

After thorough analysis of the reported strength data in all included studies, the results for lower extremity strength (leg press and extension) could be pooled from six studies [18, 20, 22, 25, 32, 41]. Test for heterogeneity of upper extremity strength was significant which implies heterogeneity between the studies (*Q*(*df* = 7) = 560.423, *p* < 0.0001). The mean differences as well as their standard errors are presented in Supplementary Table 4. The meta-analysis model for lower extremity strength showed significant higher strength values after RE (95% CI 23.42 [11.95, 34.88]) (Fig. 3). The funnel plot for lower extremity strength showed no sign of publication bias as the observed outcome is evenly distributed around the average (Supplementary Fig. 5).

## Discussion

To our knowledge, this is the first time that a meta-analysis pooled homogeneous BCRL outcome measures of five or more RE intervention trials with BCS. The results of this meta-analysis suggest that RE has a significant positive effect on BCRL in BCS. In the year 2000, Harris and Niesen-Vertommen [10] were the first to publicly challenge at that time the prevalent paradigm that physical loading of the affected arm could exacerbate an existing or trigger the development of a fresh BCRL in patients suffering from breast cancer. The first series of resistance exercise intervention studies enabled the publication of the first systematic reviews about this topic in the mid-2010s [12, 13] all of them concluding that RE will most probably not have a systematic negative effect on the BCRL. However, due to the absence of an LE assessment gold standard and therefore inconsistent assessment techniques, it has not been possible up-to-date to perform a thorough meta-analysis. So, to our knowledge, this is the first meta-analysis which pooled the same LE outcome parameters and showed that RE is not just not detrimental but beneficial for the BCRL.

However, those results need to be treated with caution for several reasons. First, the pooled BIS results might have been homogeneous regarding outcome assessment, but unfortunately, they were not regarding study protocol and therefore measurement times. While four of the studies were assessing acute responses of the BCRL [21, 30, 33, 42], the two others were assessing chronic responses [20, 43]. We can therefore not differentiate between short-term and long-term responses. The average effect, however, is significant.

Second, the effect size of the pooled effect is relatively small. However, as just mentioned before, the RE intervention times were heterogeneous regarding duration, and only two of the studies had RE intervention times of 12 weeks [20, 43]. It might therefore be possible that the effect becomes stronger with longer intervention times.

Third, the studies did not differentiate between patients who underwent sentinel lymph node dissection and those who received full axillary dissection. As the surgery technique and, therefore, the number of residual axillary lymph nodes might be decisive factors for the efficacy of RE on the lymphatic drainage, this differentiation should be considered in future research.

Fourth, RE intensities were mixed together starting with low intensity RE groups [20, 21, 30, 42] to moderate intensity RE groups [30, 33] to high intensity RE groups [20, 21, 30, 43] and even one very high intensity RE group [42]. We are therefore not able to differentiate between different RE intensities but on the other hand get a result which is representative for the heterogeneity of RE interventions in practice.

And fifth, the assessment technique BIS might be able to measure a patient’s total body water as well as extracellular and intracellular fluid volumes, but cannot differentiate between arm LE and arm muscle mass [53, 54]. This is particularly important in RE intervention studies as muscle can grow underneath an existing LE and more likely in the affected arm [27]. This is also an argument against assessment of BCRL with circumference measurements. Of all the included studies, twelve assessed BCRL with circumference measurements either as the sole BCRL measurement or as an additional parameter [18, 20, 21, 24, 28–33, 44, 45]. Although technically the sheer amount of results would allow conducting a meta-analysis, the results of these measurements were not used because of the various different measurement techniques which were utilized. Moreover, assessment of the arm volume alone is just not sufficient in RE intervention studies where muscle growth has to be considered, particularly in studies which focus on the chronic, long-term response of the affected arm. This specific assessment limitation might distort the results of several RE intervention studies. Ammitzbøll et al. [41], for example, measured higher arm volumes in the RE than in the control group (using water displacement). However, the results of their DXA suggested volume difference probably due to a better maintenance of muscle mass in the RE group compared to the control group [41]. Bloomquist et al. [43], on the other hand, described the point prevalence of LE defined by L-Dex values larger than ten. They reported in their HI-RE group no BCRL at baseline, but at the 12 and the 39 weeks follow-up, about 10% of the participants had L-Dex values indicating BCRL [43]. However, it is impossible to thoroughly understand these results, as in this study only LE assessment techniques were utilized which are unreliable regarding arm tissue differentiation in BCRL (BIS and DXA). Considering these limitations and the results of this meta-analysis, it is of utmost importance that in future RE intervention studies with breast cancer patients LE assessment techniques are utilized which allow for arm tissue differentiation, because it is still unclear, if the BCRL deterioration which was reported in those few patients is truly representative for the worsening of the LE or if in reality it might be a measuring error due to flawed assessment methods which do not allow for the assessment of muscle growth. This remains to be resolved in future research.

Furthermore, the results of the current meta-analysis open several new questions for future research. First, if RE might be beneficial for BCRL, which RE mode is most efficient? Which intensity? Which exercises? Second, do all BCS benefit from the same exercises? Are there maybe treatment-related factors like the number of residual axillary lymph nodes which might determine the efficacy of the RE intervention? Third, a RE intervention is a very controlled environment where patients perform cyclic contractions of predefined exercises. Can the positive results of those RE intervention studies actually be generalized to any (exhausting) physical loading of the upper extremities? Is the controlled RE environment really representative for any physical loading of the upper extremities that patients might be confronted with in their private and work environment?

The following limitations of the study have to be taken into account: First, as already mentioned, the BIS results of this meta-analysis might have been homogeneous regarding the LE assessment method but are heterogeneous regarding measurement time as well as exercise intensities. However, this form of heterogeneity of the studies used for the meta-analysis was representative for the entirety of the published literature. And second, those papers which could be utilized to show the pooled effect for upper and lower extremity strength were only partially the same papers which were used for the pooled BCRL analysis. BCRL and strength results from one and the same paper were meta-analyzed only of two studies [20, 43].

The strengths of this study include the systematic approach to the data collection and the study design as both a systematic review and a meta-analysis and therefore the comprehensive display of the results. Moreover, this article has been drafted by a group of researchers who has profound experience in conducting systematic reviews and meta-analyses in the field of exercise oncology [11, 12, 55, 56].

Nevertheless, the results of the current meta-analysis cannot be directly translated into clinical practice without taking some safety precautions. As long as it is unclear why a small number of patients experience a potentially detrimental effect of RE on their BCRL, it has to be concluded that several safety measures should always be considered before RE recommendation. First, inclusion in and clearance for RE intervention programs should always be undertaken after thorough clinical examination of a medical specialist. Second, the development of the BCRL should always be monitored during the RE intervention program. And third, this RE intervention program should be at least partially supervised by an exercise specialist.

However, considering the significant reduction of BCRL which has been shown in our respective meta-analysis model, the shift of paradigm regarding RE in patients suffering from or at risk of BCRL which has started 20 years ago, when BCS were advised to refrain from intensive loading of their affected arms, seems to have come to a complete turnaround into its opposite.

## Electronic supplementary material

Supplementary Fig. 1 Flowchart of the systematic literature research and the selection process. *ACSM* American College of Sports Medicine, *RE* Resistance Exercise, *LE* Lymphedema (JPG 105 kb)

Supplementary Fig. 2 RoB 2: Cochrane risk of bias assessment of the randomized trials included since September 30th 2017, details of previous studies published in Hasenoehrl et al. (2020) [11] and Keilani et al. (2015) [12] (JPG 62.4 kb)

Supplementary Fig. 3 Funnel plot Bioimpedance Spectroscopy (BIS). (JPG 60 kb)

Supplementary Fig. 4 Funnel plot upper extremity strength (chest press). (JPG 58 kb)

Supplementary Fig. 5 Funnel plot lower extremity strength (leg press & extension). (JPG 59 kb)

Supplementary Table 1 (DOCX 15.8 kb)

Supplementary Table 2 (DOCX 16.1 kb)

Supplementary Table 3 (DOCX 17.1 kb)

Supplementary Table 4 (DOCX 16.8 kb)

## Data Availability

This review was written complying the international guidelines of good scientific practice. As this is a review article, no primary data is available but all background information concerning the methodology of the creation of this paper is open for journal review if requested. This paper was written by an interdisciplinary team at the Department of Physical Medicine, Rehabilitation and Occupational Medicine, Medical University of Vienna, Austria. The preparation of this review took place within the scope of the regular research work.
