# Supplementary figures and images for: Resistance exercise and breast cancer–related lymphedema—a systematic review update and meta-analysis
Source: Support Care Cancer. 2020 May 15;28(8):3593–603. doi: 10.1007/s00520-020-05521-x (PMC7316683; doi:10.1007/s00520-020-05521-x)

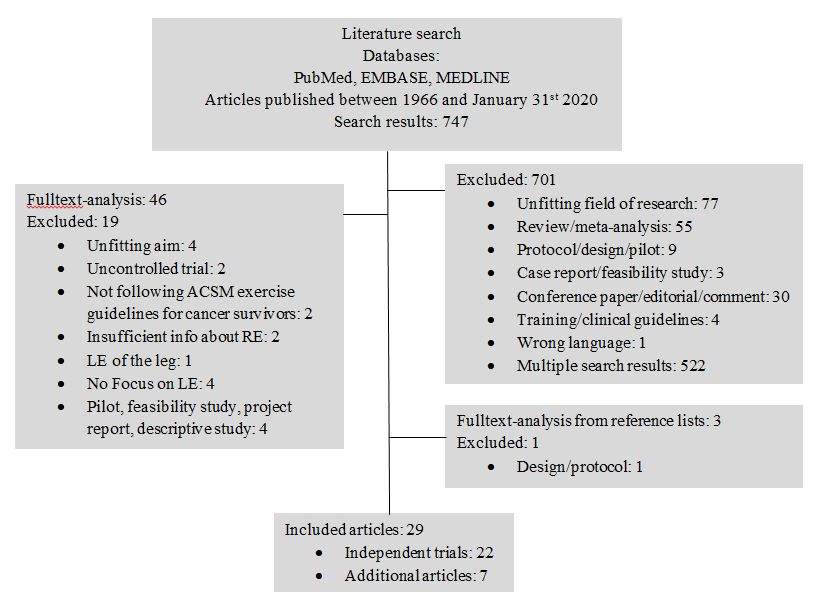

Supplement: Supplementary file 1 — Flowchart of the systematic literature research and the selection process. ACSM American College of Sports Medicine, RE Resistance Exercise, LE Lymphedema (JPG 105 kb) [file 520_2020_5521_MOESM1_ESM.jpg]

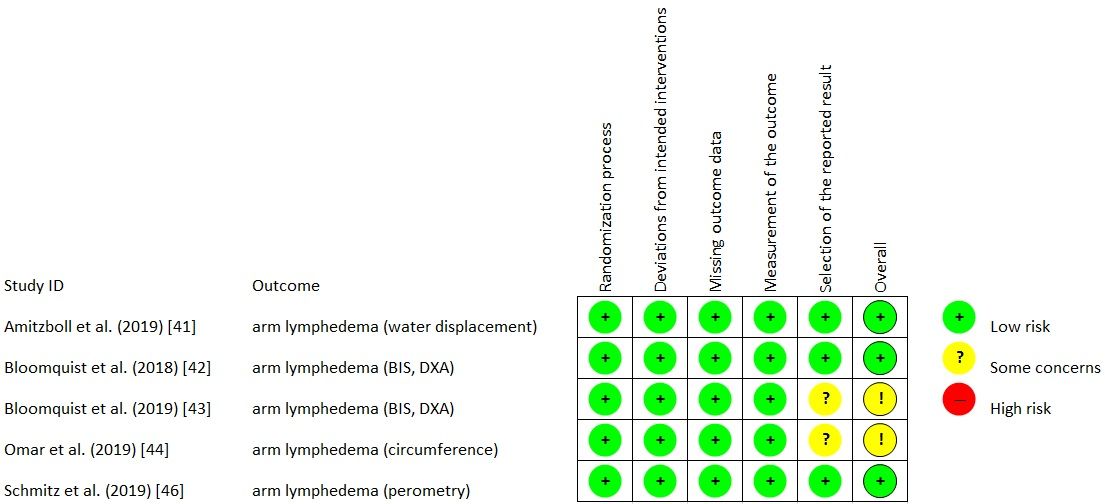

Supplement: Supplementary file 2 — RoB 2: Cochrane risk of bias assessment of the randomized trials included since September 30th 2017, details of previous studies published in Hasenoehrl et al. (2020) [11] and Keilani et al. (2015) [12] (JPG 62.4 kb) [file 520_2020_5521_MOESM2_ESM.jpg]

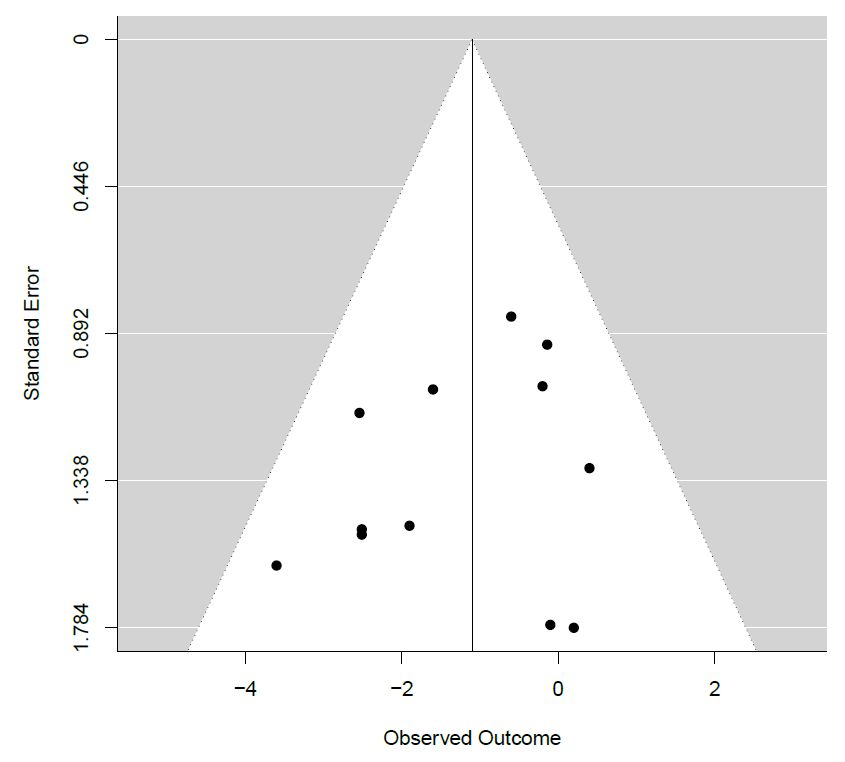

Supplement: Supplementary file 3 — Funnel plot Bioimpedance Spectroscopy (BIS). (JPG 60 kb) [file 520_2020_5521_MOESM3_ESM.jpg]

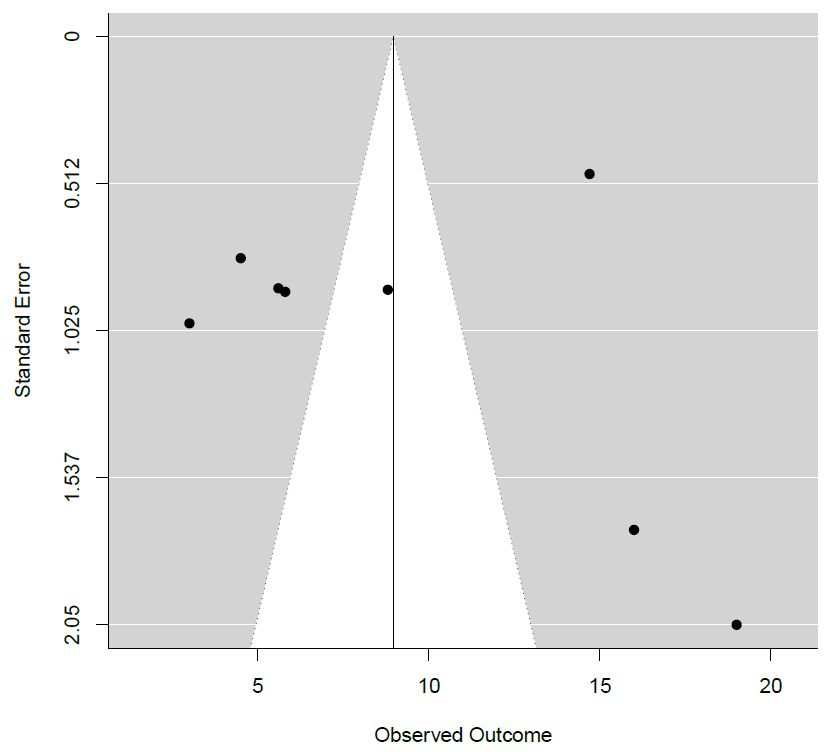

Supplement: Supplementary file 4 — Funnel plot upper extremity strength (chest press). (JPG 58 kb) [file 520_2020_5521_MOESM4_ESM.jpg]

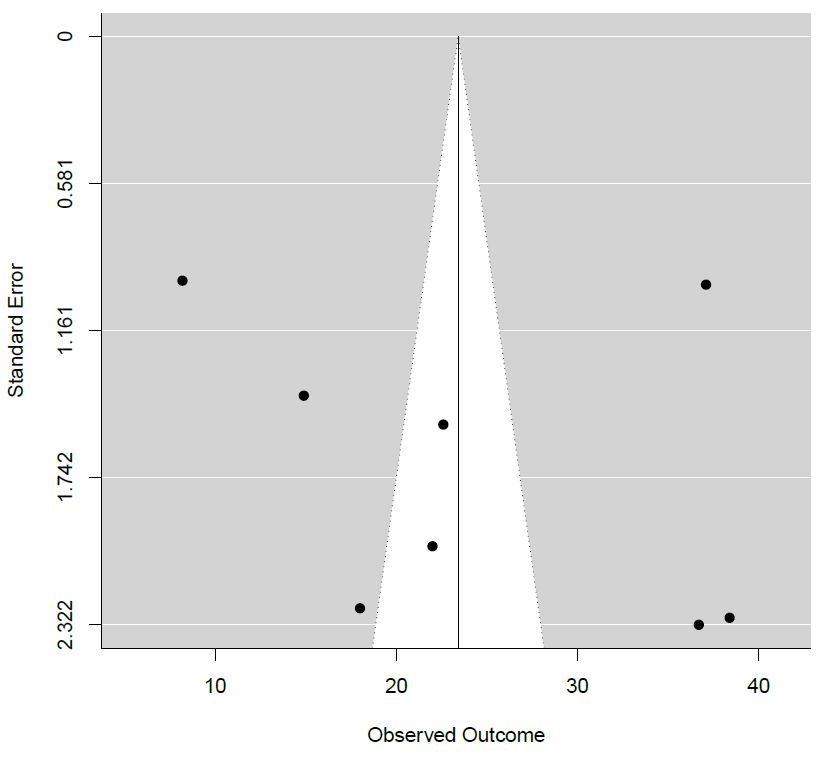

Supplement: Supplementary file 5 — Funnel plot lower extremity strength (leg press & extension). (JPG 59 kb) [file 520_2020_5521_MOESM5_ESM.jpg]
