# Supplementary material for: Resistance exercise and breast cancer–related lymphedema—a systematic review update and meta-analysis
Source: Support Care Cancer. 2020 May 15;28(8):3593–603. doi: 10.1007/s00520-020-05521-x (PMC7316683; doi:10.1007/s00520-020-05521-x)
Supplement: Supplementary file 6 — (DOCX 15.8 kb) [file 520_2020_5521_MOESM6_ESM.docx]

|  | Bias due to confounding | Bias in selection of participants into the study | Bias in classification of interventions | Bias due to deviations from intended interventions | Bias due to missing data | Bias in measurement of outcomes | Bias in selection of the reported result | Overall bias |
| --- | --- | --- | --- | --- | --- | --- | --- | --- |
| Luz et al. (2018)[45] | Serious* | Low | Low | Low | Low | Moderate** | Low | Serious |
| Explanation:  * BMI and lymphedema stage were different between the intervention groups at baseline  ** The outcome assessors aware of the intervention received; outcome measure could have been influenced by knowledge of the intervention received | | | | | | | | |

Supplementary Table 1. Risk of bias assessment of the included non-randomized study (Luz et al., 2018) [45]
