# Supplementary material for: Resistance exercise and breast cancer–related lymphedema—a systematic review update and meta-analysis
Source: Support Care Cancer. 2020 May 15;28(8):3593–603. doi: 10.1007/s00520-020-05521-x (PMC7316683; doi:10.1007/s00520-020-05521-x)
Supplement: Supplementary file 7 — (DOCX 16.1 kb) [file 520_2020_5521_MOESM7_ESM.docx]

| Study | Subgroup | Mean difference | SE |
| --- | --- | --- | --- |
| Cormie et al. (2013) [20] | HI-RE | -0.20 | 1.11 |
| Cormie et al. (2013) [20] | LI-RE | -1.60 | 1.13 |
| Cormie et al. (2013a) [21] | HI-RE | 0.20 | 3.18 |
| Cormie et al. (2013a) [21] | LI-RE | -0.10 | 3.15 |
| Cormie et al. (2016) [30] | HI-RE | -2.54 | 1.28 |
| Cormie et al. (2016) [30] | MI-RE | -2.51 | 2.21 |
| Cormie et al. (2016) [30] | LI-RE | -2.51 | 2.26 |
| Singh et al. (2015) [33] | MI-RE Compr | -3.60 | 2.55 |
| Singh et al. (2015) [33] | MI-RE No-Compr | -1.90 | 2.18 |
| Bloomquist et al. (2018) [42] | (very) HI-RE | -0.14 | 0.86 |
| Bloomquist et al. (2018) [42] | LI-RE | -0.60 | 0.71 |
| Bloomquist et al. (2019) [43] | HI-RE | 0.40 | 1.69 |

Supplementary Table 2. Mean differences and SE of the study subgroups pooled for meta-analysis of the bioimpedance spectroscopy (BIS) results
