# Supplementary material for: Resistance exercise and breast cancer–related lymphedema—a systematic review update and meta-analysis
Source: Support Care Cancer. 2020 May 15;28(8):3593–603. doi: 10.1007/s00520-020-05521-x (PMC7316683; doi:10.1007/s00520-020-05521-x)
Supplement: Supplementary file 8 — (DOCX 17.1 kb) [file 520_2020_5521_MOESM8_ESM.docx]

| Study | Subgroup | Mean difference | SE |
| --- | --- | --- | --- |
| Cormie et al. (2013) [20] | HI-RE | 5.60 | 0.77 |
| Cormie et al. (2013) [20] | LI-RE | 5.80 | 0.79 |
| Courneya et al. (2007) [22] | MI-RE | 8.80 | 0.78 |
| Schmitz et al. (2009) [25] | MI-RE | 4.50 | 0.60 |
| Ahmed et al. (2006) [18] | MI-RE | 14.70 | 0.23 |
| Simonavice et al. (2014) [32] | MI-RE | 16.00 | 2.96 |
| Simonavice et al. (2014) [32] | MI-RE + suppl | 19.00 | 4.20 |
| Bloomquist et al. (2019) [43] | HI-RE | 3.00 | 1.00 |

Supplementary Table 3. Mean differences and SE of the study subgroups pooled for meta-analysis of the upper extremity strength results (chest press)
