# Supplementary material for: Resistance exercise and breast cancer–related lymphedema—a systematic review update and meta-analysis
Source: Support Care Cancer. 2020 May 15;28(8):3593–603. doi: 10.1007/s00520-020-05521-x (PMC7316683; doi:10.1007/s00520-020-05521-x)
Supplement: Supplementary file 9 — (DOCX 16.8 kb) [file 520_2020_5521_MOESM9_ESM.docx]

| Study | Subgroup | Mean difference | SE |
| --- | --- | --- | --- |
| Cormie et al. (2013) [20] | HI-RE | 38.40 | 5.27 |
| Cormie et al. (2013) [20] | LI-RE | 36.70 | 5.39 |
| Courneya et al. (2007) [22] | MI-RE | 8.20 | 0.93 |
| Schmitz et al. (2009) [25] | MI-RE | 22.60 | 2.35 |
| Ahmed et al. (2006) [18] | MI-RE | 37.10 | 0.96 |
| Simonavice et al. (2014) [32] | MI-RE | 18.00 | 5.10 |
| Simonavice et al. (2014) [32] | MI-RE + suppl | 22.00 | 4.05 |
| Ammitzboll et al. (2019) [41] | MI-RE | 14.90 | 2.01 |

Supplementary Table 4. Mean differences and SE of the study subgroups pooled for meta-analysis of the lower extremity strength results (leg press & extension)
